# 

**Published:** 1900-04

**Authors:** 


### MARRIAGE.

W. H. Derr, V.S., graduate of Ontario Veterinary College, 1893, to Miss Elizabeth Tulloss, both of Mansfield, Ohio. The recently married couple are at home to their friends on E. Fourth Street after April 1.

### ASSOCIATION MEETINGS.

THE regular meeting of the Veterinary Medical Association of New Jersey will be held at Jacoby's, 882 Broad Street, Newark, N. J., Thursday, May 10th, at 10 A.M.

S. LOCKWOOD,
Secretary.

### THE NIAGARA AND ORLEANS COUNTY VETERINARY MEDICAL SOCIETY.

An organization meeting was held on April 14, 1900, in the parlors of the Rich Hotel, Middleport, N. Y. Drs. Stocking, Williams, and Crowforth were appointed essayists for the next meeting of the Society, which will be held in Lockport, N. Y., August 11, 1900, and will be known as the annual meeting.

Anderson Crowforth,
Secretary-Treasurer.

### PERSONALS.

Dr. F. T. Shannon, graduate of the Veterinary Department of the University of Pennsylvania, an attaché of the Bureau of Animal Industry, recently located in Southern California, and now connected with the pathological department of the bureau at Washington, was a visitor to Philadelphia in March.

Dr. W. H. Pendry, of Brooklyn, N. Y., has been suffering for more than three weeks, but is now again in harness.

Dr. E. P. Flowers, of Dallas, Texas, is taking a post-graduate course at the University of Pennsylvania Veterinary Department.

Professor Andrew Smith, of Toronto, has been selected as President of the Industrial Fair to be held in that city in September next.

Dr. E. P. Spaeth, of Gillette, Wyoming, was a visitor to Philadelphia in March, and addressed the Students' Society of the Veterinary Department on "Sheep Raising."

Drs. John W. Adams and E. M. Ranck, of Philadelphia, were severe sufferers from influenza in March, and were confined to their rooms for a number of days.

Dr. William Dougherty, of Baltimore, Md., was off duty for several weeks in March. Dr. Martinet looked after the doctor's practice while ill.

Dr. W. S. Manley, of Pittsburg, Kansas, was a visitor to the Kansas City and Western Veterinary Colleges in March, attending the closing exercises.

Dr. George Fuller, of Marshall & Fuller, veterinarians, of Philadelphia, was on the sick list for two weeks in March.

Dr. E. E. Sayers continues to fill the all-important post of Mayor of his city, Algona, Iowa.

Dr. James B. Rayner, of West Chester, Pa., was confined to his house for two weeks in March.

Dr. H. D. Gill, of New York City, is building a very complete equine and canine hospital, residence and laboratories combined.

Dr. George B. Jobson's subject at the Pennsylvania Jersey Cattle Breeders' Association was entitled "A Healthy Herd, and How to Keep it So." State Veterinarian Pearson addressed the same body on the importance and value of "Certified Milk."

Dr. T. Delaney, of New York City, was a purchaser of one of the choice pairs of show horses at the recent sale of the estate of C. F. Bates.

Dr. Guildin R. Hartman, of Philadelphia, milk inspector of the Board of Health, was arrested in the latter part of March, charged by a city dairyman with extorting money to suppress information in his possession of a violation of the milk ordinances of this city.

Dr. William H. Lowe, of Paterson, N. J., takes an active part in city affairs, and is ever ready to lend a helping hand for what is for the welfare of his city.

Dr. W. S. Kooker, of Philadelphia, has been in poor health all winter, due to digestive derangement.

Dr. Harry Walters, formerly of Wilkesbarre, has located in Trenton, N. J., where he is conducting a sanitarium for nervous diseases of the human family.

Dr. W. Horace Hoskins, of Philadelphia, was a visitor to New York in March, in connection with matters pertaining to the welfare of his alma mater.

Dr. Walter L. Burt, formerly of Providence, R. I., now of Port Chester, N. Y., gives his entire attention to the purchase and sale of high-grade coach and driving horses.

Dr. William Dimond, of Newark, N. J., takes an active part in municipal affairs, and is the candidate of the Democratic party for membership on the Board of Public Works.

Dr. S. E. Weber, of Green Lane, has entered the sanitarium of Drs. Walters and Arnold at Trenton, N. J.

Professor Wesley Mills, of Montreal, Canada, is spending some time in Germany.

Dr. A. J. Tuxill, of Auburn, N. Y., is rapidly regaining his old-time health and strength after a long period of illness.

Dr. J. Stewart Lacock, of Allegheny, Pa., fills the rôle of veterinarian to the Duquesne Kennel Club of Western Pennsylvania. The club holds its show the latter part of April.

Dr. Benjamin A. Harris, of Boston, Mass., has entered the commercial world and engaged in the sale of horse clothing and stable equipments.

Dr. Levi Collner, of St. Petersburg, Pa., has been on the sick list for a time.

Dr. W. F. Smith, of Amsterdam, N. Y., has resigned his position as manager of the Hurricane Stud Farm, to locate in England.

Dr. Joseph Heagerty has become the city veterinarian for Baltimore, Md., having received the appointment at the hands of Mayor Hayes, who was recently installed as Chief Magistrate of the "Oriole City."

Dr. W. N. D. Bird, of the Bureau of Animal Industry, has been transferred from St. Louis, Missouri, to East Buffalo, N. Y.

Dr. H. E. Wand, of Ambler, Pa., has changed his location, and rumor has it that he has gone to Old England.

Dr. W. Horace Hoskins, of Philadelphia, represents one of the city districts on the State Central Democratic Committee.

Dr. Charles Gresswell, of Denver, Col., in addition to his professional work, is engaged extensively with others in the cattle trade.

Mayor Ashbridge, of Philadelphia, has failed to reappoint Dr. John W. Adams, consulting city veterinarian, at the expiration of his term on April 1. It will be well to note the attitude of the Chief Magistrate of the "City of Brotherly Love" in his selection of a successor to Dr. Adams.

Dr. E. M. Ranck, of Philadelphia, has been doing some special work for the State Livestock Sanitary Board among cattle in the neighborhood of his former home in Lancaster County, Pa.

Dr. Stephen L. Blount, of Temple, Tex., was among the applicants for meat inspector at the April examination.

Dr. W. E. D. Rushworth, of Buffalo, formerly of the meat-inspection service, has become assistant manager to the Pasteur Vaccine Company, and with his knowledge of sheep and among sheepmen will be of much value to them in their field of work.

Dr. R. C. Moore, of the Kansas City Veterinary College, was recently appointed assistant State Veterinarian of Missouri.

Drs. S. D. Larzelere, of Jenkintown, Pa.; A. T. Sellers, of Camden, N. J., and W. Horace Hoskins, of Philadelphia, were among the Easter boardwalk visitors at Atlantic City, N. J.

Brewer Nolan, the pet of the senior class of the Veterinary Department of the University of Pennsylvania, tarried long beyond the allotted time from class duties at "Eastertide." Dame rumor has it that the attraction of Easter novelties and costumes among the fair ones of the "Oriole City" tells the tale.

Dr. P. M. Minster, of Langhorne, Pa., finds the exacting duties of his profession, his drug store exactions, and as president of the local board of health, a severe strain on his physical condition.

Dr. Leonard Pearson, of Philadelphia, was a visitor to Boston, Mass., in April, paying a visit to the Veterinary Department of Harvard University.

Dr. William H. Martinet, of Baltimore, is likely to secure the position of Assistant State Veterinarian of Maryland.

Dr. James Beatty, of the Quarantine Service of the Bureau of Animal Industry, has been transferred to "Greater New York," where he will find a more congenial location.

Dr. B. F. Minnich, of Columbia, Pa., is an admirer of the trotting-horse, and recently added an "Allerton" colt to his list of roadsters.

Dr. C. E. Clayton, assistant-surgeon of the New York American Veterinary College Hospital, has severed his connection therewith and entered upon private practice in New York City.

Mr. L. A. Nolan, senior student of the Veterinary Department of the University of Pennsylvania, is a great admirer of the mastiff variety of canines.

Dr. A. D. Eastman, of East Trumbull, Ohio, is a breeder of registered Jersey cattle and Chester White swine.

Dr. J. Stewart Lacock, of Pittsburg, finds in the dog shows of the land a field of pleasure and delight as he notes the added ribbons to his petted companions.

